# (Anilino{(*Z*)-2-[(*E*)-5-bromo-3-meth­oxy-2-oxidobenzyl­idene]hydrazin-1-yl­idene-κ^2^ *O* ^2^,*N* ^2^}methane­thiol­ato-κ*S*)(4,4′-di­methyl-2,2′-bipyridine-κ^2^ *N*,*N*′)zinc *N*,*N*-dimethyl­formamide monosolvate

**DOI:** 10.1107/S1600536812031777

**Published:** 2012-07-18

**Authors:** Jinsa Mary Jacob, M. R. Prathapachandra Kurup, Seik Weng Ng

**Affiliations:** a Department of Applied Chemistry, Cochin University of Science and Technology, Kochi 682 022, India; b Department of Chemistry, University of Malaya, 50603 Kuala Lumpur, Malaysia; c Chemistry Department, King Abdulaziz University, PO Box 80203 Jeddah, Saudi Arabia

## Abstract

The asymmetric unit of the title compound, [Zn(C_15_H_12_BrN_3_O_2_S)(C_12_H_12_N_2_)]·C_3_H_7_NO, contains two independent mol­ecules with a similar structure. The doubly deprotonated Schiff base ligand *O*,*N*,*S*-chelates to the metal atom, and the three coordinating atoms along with one N atom of the substituted 2,2′-bipyridine ligand constitute the square plane of the distorted square pyramid surrounding the metal atom. The apical site is occupied by the second N atom of the substituted 2,2′-bipyridine. The secondary amine group of the Schiff base dianion forms a hydrogen bond to the O atom of the dimethyl­formamide solvent. In the crystal, the phenyl ring of one of the two Schiff base anions is disordered over two positions in a 1:1 ratio. The crystal studied is a racemic twin.

## Related literature

For a related zinc structure, see: Seena & Kurup (2008 ▶).
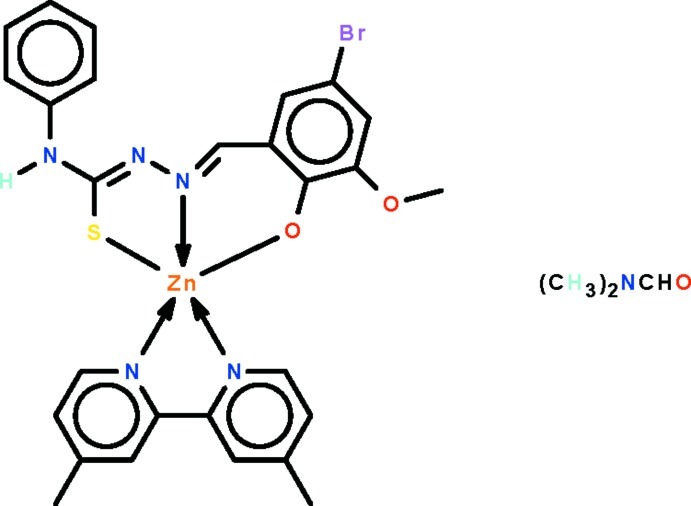


## Experimental

### 

#### Crystal data

[Zn(C_15_H_12_BrN_3_O_2_S)(C_12_H_12_N_2_)]·C_3_H_7_NO
*M* *_r_* = 700.95
Monoclinic, 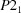

*a* = 15.2674 (3) Å
*b* = 12.2422 (3) Å
*c* = 22.3402 (5) Å
β = 131.425 (1)°
*V* = 3130.90 (12) Å^3^
*Z* = 4
Mo *K*α radiation
μ = 2.17 mm^−1^
*T* = 293 K
0.40 × 0.30 × 0.25 mm

#### Data collection

Bruker APEXII diffractometer
Absorption correction: multi-scan (*SADABS*; Sheldrick, 1996 ▶) *T*_min_ = 0.478, *T*_max_ = 0.613
52133 measured reflections
14238 independent reflections
9143 reflections with *I* > 2σ(*I*)
*R*_int_ = 0.051

#### Refinement

*R*[*F* ^2^ > 2σ(*F* ^2^)] = 0.070
*wR*(*F* ^2^) = 0.211
*S* = 1.01
14238 reflections
676 parameters
66 restraints
H-atom parameters constrained
Δρ_max_ = 1.22 e Å^−3^
Δρ_min_ = −1.64 e Å^−3^
Absolute structure: Flack (1983 ▶), 6722 Friedel pairs
Flack parameter: 0.50 (2)

### 

Data collection: *APEX2* (Bruker, 2004 ▶); cell refinement: *SAINT* (Bruker, 2004 ▶); data reduction: *SAINT*; program(s) used to solve structure: *SHELXS97* (Sheldrick, 2008 ▶); program(s) used to refine structure: *SHELXL97* (Sheldrick, 2008 ▶); molecular graphics: *X-SEED* (Barbour, 2001 ▶); software used to prepare material for publication: *publCIF* (Westrip, 2010 ▶).

## Supplementary Material

Crystal structure: contains datablock(s) global, I. DOI: 10.1107/S1600536812031777/xu5589sup1.cif

Structure factors: contains datablock(s) I. DOI: 10.1107/S1600536812031777/xu5589Isup2.hkl

Additional supplementary materials:  crystallographic information; 3D view; checkCIF report

## Figures and Tables

**Table 1:** Hydrogen-bond geometry (Å, °)

*D*—H⋯*A*	*D*—H	H⋯*A*	*D*⋯*A*	*D*—H⋯*A*
N3—H3⋯O5	0.88	2.07	2.95 (1)	175
N8—H8⋯O6	0.88	2.07	2.95 (1)	172

**Fig. 1.**
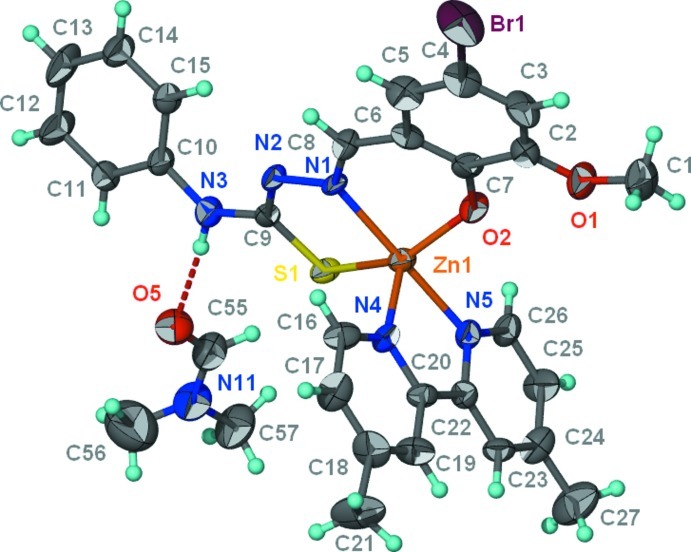
Thermal ellipsoid plot (Barbour, 2001) of one Zn(C12H12N2)(C15H12BrN3O2S).DMF molecule at the 50% probability level; hydrogen atoms are drawn as spheres of arbitrary radius. The disorder in one of the phenyl rings is not shown.

**Fig. 2.**
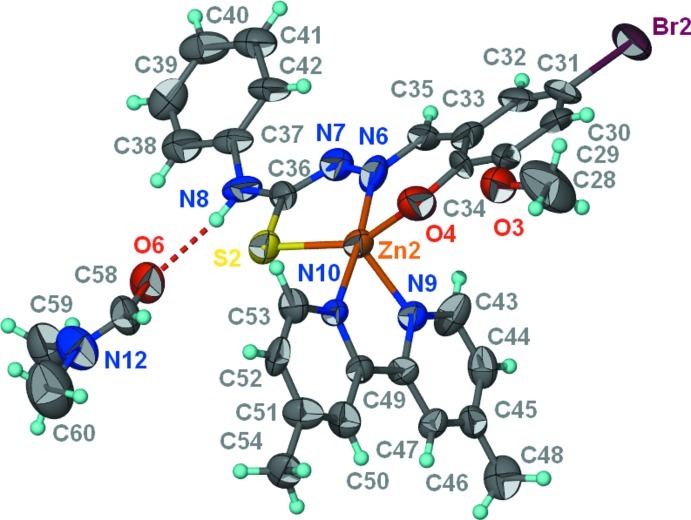
Thermal ellipsoid plot (Barbour, 2001) of second Zn(C12H12N2)(C15H12BrN3O2S).DMF molecule at the 50% probability level; hydrogen atoms are drawn as spheres of arbitrary radius. The disorder in one of the phenyl rings is not shown

## supplementary crystallographic information

### Comment

A large number of first-row transition metal derivatives of Schiff bases that are synthesized by reacting salicylaldehyde-type of aldehydes with 4-phenylthiosemicarbazide has been reported. The zinc homolog has been isolated as a 2,2'-bipyridine adduct (Seena & Kurup, 2008). The metal center shows square-pyramidal coordination as one of the pyridine N atoms occupy the apical site. Substituents in the Schiff base as well as in the 2,2'-bipyridine do not perturb the square pyramidal coordination geometry in Zn(C_12_H_12_N_2_)(C_15_H_12_BrN_3_O_2_S)^.^DMF (Scheme I). The compound crystallizes as a DMF solvate (Figs. 1 and 2).

The doubly-deprotonated Schiff base in *O*,*N*,*S*-chelates to the metal atom, and the three coordinating atoms along with the N atom of the substituted bipyridine ligand comprise the square plane of the square pyramid surrounding it. The apical site is occupied by the second N atom of the substituted 2,2'-bipyridine. In one molecule, the Zn is displaced by 0.305 (3) Å in the direction of the apical occupant (Fig. 1) whereas in the other, the displacement is 0.103 (6) Å in the opposite direction (Fig. 2). The secondary amino group of the Schiff-base dianion forms a hydrogen bond to the O atom of the DMF (Table 1).

### Experimental

To a stirred mixture of 2-(5-bromo-2-hydroxy-3-methoxybenzylidene)-*N*-phenylhydrazinecarbothioamide (0.190 g, 0.5 mmol) in a 1:1 mixture of DMF and methanol and 4,4'-dimethyl-2,2'-bipyridine (0.092 g, 0.5 mmol) in methanol, zinc(II) acetate dihydrate (0.109 g, 0.5 mmol) was added. The resulting yellow solution was heated for 3 h. Yellow crystals separated from the solution after several days.

### Refinement

Carbon- and nitrogen bound H-atoms were placed in calculated positions (C–H 0.93 to 0.96 Å, N–H 0.88 Å) and were included in the refinement in the riding model approximation, with *U*(H) set to 1.2 to 1.5*U*(C,*N*).

Omitted owing to bad disagreement was (0 1 1).

All aromatic and pyridine rings were refined as rigid hexagons of 1.39 Å sides. One of the phenyl rings of the Schiff-base anion is disordered over two positions in a 1:1 ratio. The temperature factors of the primed atoms were set to those of the unprimed ones but in the reverse order (*i.e.*, those of C11 to those of C15), and the pair of *N*–C_phenyl_ distances were restrained to within 0.01 Å of each other.

The molecules of DMF were each restrained to lie on a plane; their. The anisotropic temperature factors were restrained to be nearly isotropic.

The final difference Fourier map had a peak at 0.91 Å from Br1 and a hole at 0.96 Å from Br2.

The base scale factor was explicitly refined.

### Crystal data

**Table:**

[Zn(C_15_H_12_BrN_3_O_2_S)(C_12_H_12_N_2_)]·C_3_H_7_NO	*F*(000) = 1432
*M**_r_* = 700.95	*D*_x_ = 1.487 Mg m^−^^3^
Monoclinic, *P*2_1_	Mo *K*α radiation, λ = 0.71073 Å
Hall symbol: P 2yb	Cell parameters from 9863 reflections
*a* = 15.2674 (3) Å	θ = 2.5–26.3°
*b* = 12.2422 (3) Å	µ = 2.17 mm^−^^1^
*c* = 22.3402 (5) Å	*T* = 293 K
β = 131.425 (1)°	Prism, yellow
*V* = 3130.90 (12) Å^3^	0.40 × 0.30 × 0.25 mm
*Z* = 4	

### Data collection

**Table:**

Bruker APEXII diffractometer	14238 independent reflections
Radiation source: fine-focus sealed tube	9143 reflections with *I* > 2σ(*I*)
Graphite monochromator	*R*_int_ = 0.051
ω scans	θ_max_ = 27.5°, θ_min_ = 1.2°
Absorption correction: multi-scan (*SADABS*; Sheldrick, 1996)	*h* = −19→19
*T*_min_ = 0.478, *T*_max_ = 0.613	*k* = −15→15
52133 measured reflections	*l* = −29→28

### Refinement

**Table:**

Refinement on *F*^2^	Secondary atom site location: difference Fourier map
Least-squares matrix: full	Hydrogen site location: inferred from neighbouring sites
*R*[*F*^2^ > 2σ(*F*^2^)] = 0.070	H-atom parameters constrained
*wR*(*F*^2^) = 0.211	*w* = 1/[σ^2^(*F*_o_^2^) + (0.0841*P*)^2^ + 9.813*P*] where *P* = (*F*_o_^2^ + 2*F*_c_^2^)/3
*S* = 1.01	(Δ/σ)_max_ = 0.001
14238 reflections	Δρ_max_ = 1.22 e Å^−^^3^
676 parameters	Δρ_min_ = −1.64 e Å^−^^3^
66 restraints	Absolute structure: Flack (1983), 6722 Friedel pairs
Primary atom site location: structure-invariant direct methods	Flack parameter: 0.50 (2)

### Fractional atomic coordinates and isotropic or equivalent isotropic displacement parameters (Å^2^)

**Table:**

	*x*	*y*	*z*	*U*_iso_*/*U*_eq_	Occ. (<1)
Br1	1.72718 (11)	−0.00210 (15)	1.13837 (9)	0.0914 (5)	
Br2	1.72867 (13)	0.40505 (18)	0.63979 (13)	0.1416 (9)	
Zn1	1.12332 (8)	0.22235 (8)	0.86185 (6)	0.0366 (2)	
Zn2	1.12382 (11)	0.18373 (10)	0.36192 (8)	0.0669 (4)	
S1	1.02546 (19)	0.3730 (2)	0.85966 (14)	0.0399 (5)	
S2	1.0257 (3)	0.0344 (3)	0.35998 (18)	0.0745 (9)	
O1	1.3542 (6)	0.0420 (9)	0.8358 (4)	0.074 (3)	
O2	1.2468 (5)	0.1673 (6)	0.8630 (3)	0.0452 (15)	
O3	1.3522 (8)	0.3694 (10)	0.3332 (6)	0.096 (3)	
O4	1.2447 (8)	0.2454 (9)	0.3637 (6)	0.098 (3)	
O5	0.8524 (6)	0.5244 (8)	0.8923 (4)	0.066 (2)	
O6	0.8553 (8)	−0.1344 (9)	0.3904 (5)	0.090 (3)	
N1	1.2588 (5)	0.2765 (7)	0.9809 (4)	0.0347 (16)	
N2	1.2354 (5)	0.3571 (6)	1.0123 (4)	0.0353 (15)	
N3	1.1036 (6)	0.4821 (7)	0.9878 (4)	0.0450 (19)	
H3	1.0279	0.4927	0.9571	0.054*	0.50
H3'	1.0283	0.4963	0.9555	0.054*	0.50
N4	1.0594 (4)	0.0801 (4)	0.8728 (3)	0.0368 (17)	
C16	1.1041 (4)	0.0322 (5)	0.9445 (2)	0.049 (2)	
H16	1.1681	0.0632	0.9927	0.058*	
C17	1.0531 (6)	−0.0620 (5)	0.9444 (3)	0.060 (3)	
H17	1.0830	−0.0940	0.9924	0.071*	
C18	0.9574 (6)	−0.1083 (5)	0.8724 (4)	0.061 (3)	
C19	0.9127 (5)	−0.0604 (5)	0.8006 (3)	0.055 (3)	
H19	0.8486	−0.0914	0.7525	0.066*	
C20	0.9637 (5)	0.0337 (4)	0.8008 (2)	0.0382 (19)	
N5	0.9842 (4)	0.1788 (5)	0.7400 (2)	0.0400 (17)	
C22	0.9212 (5)	0.0881 (4)	0.7305 (3)	0.039 (2)	
C23	0.8202 (5)	0.0574 (4)	0.6547 (3)	0.044 (2)	
H23	0.7781	−0.0033	0.6483	0.053*	
C24	0.7822 (4)	0.1174 (5)	0.5884 (2)	0.055 (3)	
C25	0.8451 (5)	0.2081 (5)	0.5979 (3)	0.066 (3)	
H25	0.8197	0.2482	0.5535	0.079*	
C26	0.9461 (5)	0.2388 (4)	0.6737 (3)	0.047 (2)	
H26	0.9882	0.2995	0.6801	0.057*	
N6	1.2560 (10)	0.1274 (9)	0.4763 (7)	0.080 (3)	
N7	1.2368 (9)	0.0517 (10)	0.5138 (6)	0.085 (3)	
N8	1.1032 (9)	−0.0762 (10)	0.4868 (6)	0.073 (3)	
H8	1.0276	−0.0868	0.4566	0.088*	
N9	1.0634 (5)	0.3279 (5)	0.3726 (4)	0.059 (2)	
C43	1.1100 (5)	0.3757 (6)	0.4448 (3)	0.085 (4)	
H43	1.1769	0.3467	0.4925	0.102*	
C44	1.0566 (6)	0.4668 (6)	0.4458 (3)	0.072 (4)	
H44	1.0878	0.4988	0.4941	0.086*	
C45	0.9566 (6)	0.5102 (5)	0.3745 (4)	0.063 (3)	
C46	0.9099 (5)	0.4624 (5)	0.3022 (3)	0.051 (2)	
H46	0.8430	0.4914	0.2545	0.061*	
C47	0.9633 (5)	0.3712 (5)	0.3013 (3)	0.049 (2)	
N10	0.9844 (4)	0.2266 (6)	0.2407 (3)	0.0542 (19)	
C49	0.9190 (5)	0.3154 (5)	0.2305 (3)	0.051 (2)	
C50	0.8194 (6)	0.3466 (5)	0.1543 (4)	0.067 (3)	
H50	0.7757	0.4061	0.1475	0.080*	
C51	0.7853 (5)	0.2890 (6)	0.0882 (3)	0.069 (3)	
C52	0.8507 (6)	0.2001 (6)	0.0983 (3)	0.065 (3)	
H52	0.8278	0.1616	0.0541	0.078*	
C53	0.9502 (6)	0.1689 (5)	0.1746 (4)	0.073 (3)	
H53	0.9940	0.1095	0.1814	0.087*	
N11	0.6681 (7)	0.5698 (6)	0.7784 (5)	0.072 (3)	
N12	0.6763 (11)	−0.1722 (9)	0.2820 (7)	0.103 (4)	
C1	1.3953 (11)	−0.0425 (12)	0.8147 (8)	0.085 (4)	
H1A	1.3449	−0.0467	0.7576	0.127*	
H1B	1.3950	−0.1113	0.8352	0.127*	
H1C	1.4733	−0.0257	0.8371	0.127*	
C2	1.4122 (5)	0.0630 (6)	0.9134 (3)	0.050 (2)	
C3	1.5234 (5)	0.0242 (5)	0.9767 (4)	0.062 (3)	
H3A	1.5613	−0.0219	0.9669	0.074*	
C4	1.5781 (4)	0.0542 (6)	1.0544 (3)	0.055 (3)	
C5	1.5216 (5)	0.1230 (6)	1.0690 (3)	0.055 (3)	
H5	1.5582	0.1431	1.1210	0.066*	
C6	1.4103 (4)	0.1619 (5)	1.0057 (3)	0.040 (2)	
C7	1.3556 (4)	0.1319 (5)	0.9279 (3)	0.051 (3)	
C8	1.3598 (8)	0.2388 (9)	1.0255 (5)	0.042 (2)	
H8A	1.4094	0.2629	1.0782	0.051*	
C9	1.1356 (8)	0.3981 (7)	0.9630 (6)	0.039 (2)	
C10	1.1736 (15)	0.5524 (14)	1.0541 (8)	0.043 (2)	0.50
C11	1.1131 (11)	0.622 (2)	1.0646 (13)	0.043 (4)	0.50
H11	1.0322	0.6178	1.0308	0.051*	0.50
C12	1.1735 (18)	0.699 (2)	1.1259 (17)	0.083 (7)	0.50
H12	1.1330	0.7457	1.1329	0.100*	0.50
C13	1.2944 (18)	0.7056 (18)	1.1765 (13)	0.080 (6)	0.50
H13	1.3348	0.7569	1.2175	0.096*	0.50
C14	1.3550 (10)	0.6357 (15)	1.1659 (9)	0.060 (4)	0.50
H14	1.4359	0.6402	1.1998	0.072*	0.50
C15	1.2946 (15)	0.5591 (11)	1.1047 (10)	0.043 (4)	0.50
H15	1.3351	0.5123	1.0976	0.052*	0.50
C10'	1.1748 (15)	0.5472 (15)	1.0570 (8)	0.043 (2)	0.50
C11'	1.1224 (10)	0.635 (2)	1.0620 (11)	0.043 (4)	0.50
H11'	1.0431	0.6488	1.0207	0.052*	0.50
C12'	1.1883 (17)	0.7029 (19)	1.1285 (14)	0.060 (4)	0.50
H12'	1.1532	0.7618	1.1318	0.072*	0.50
C13'	1.3067 (17)	0.6826 (18)	1.1902 (10)	0.080 (6)	0.50
H13'	1.3508	0.7279	1.2347	0.096*	0.50
C14'	1.3592 (11)	0.5946 (16)	1.1853 (8)	0.083 (7)	0.50
H14'	1.4384	0.5810	1.2265	0.100*	0.50
C15'	1.2933 (16)	0.5269 (10)	1.1187 (10)	0.043 (4)	0.50
H15'	1.3284	0.4680	1.1154	0.051*	0.50
C21	0.8951 (12)	−0.2053 (11)	0.8731 (8)	0.089 (4)	
H21A	0.8491	−0.1801	0.8855	0.134*	
H21B	0.9519	−0.2571	0.9127	0.134*	
H21C	0.8450	−0.2395	0.8215	0.134*	
C27	0.6743 (14)	0.0869 (17)	0.5059 (7)	0.101 (5)	
H27A	0.6592	0.0103	0.5042	0.152*	
H27B	0.6850	0.1026	0.4690	0.152*	
H27C	0.6093	0.1282	0.4916	0.152*	
C28	1.3964 (16)	0.454 (2)	0.3172 (13)	0.170 (11)	
H28A	1.3466	0.4635	0.2605	0.255*	
H28B	1.4737	0.4350	0.3390	0.255*	
H28C	1.3992	0.5202	0.3412	0.255*	
C29	1.4093 (6)	0.3422 (7)	0.4112 (4)	0.092 (5)	
C30	1.5205 (6)	0.3817 (6)	0.4740 (6)	0.096 (5)	
H30	1.5574	0.4282	0.4637	0.115*	
C31	1.5764 (5)	0.3518 (8)	0.5520 (5)	0.095 (5)	
C32	1.5212 (7)	0.2824 (8)	0.5673 (4)	0.094 (5)	
H32	1.5587	0.2624	0.6195	0.112*	
C33	1.4100 (7)	0.2428 (7)	0.5046 (5)	0.080 (4)	
C34	1.3541 (5)	0.2728 (7)	0.4266 (4)	0.071 (3)	
C35	1.3670 (10)	0.1683 (13)	0.5301 (8)	0.086 (4)	
H35	1.4152	0.1478	0.5834	0.103*	
C36	1.1316 (9)	0.0013 (10)	0.4594 (6)	0.059 (3)	
C37	1.1745 (6)	−0.1450 (7)	0.5566 (4)	0.064 (3)	
C38	1.1170 (5)	−0.2253 (8)	0.5633 (5)	0.081 (4)	
H38	1.0359	−0.2292	0.5258	0.098*	
C39	1.1807 (9)	−0.2998 (7)	0.6258 (6)	0.091 (4)	
H39	1.1423	−0.3536	0.6302	0.110*	
C40	1.3020 (9)	−0.2940 (7)	0.6817 (5)	0.095 (5)	
H40	1.3446	−0.3438	0.7236	0.114*	
C41	1.3595 (5)	−0.2136 (8)	0.6751 (4)	0.086 (4)	
H41	1.4406	−0.2097	0.7125	0.103*	
C42	1.2958 (6)	−0.1391 (7)	0.6126 (5)	0.075 (3)	
H42	1.3342	−0.0854	0.6081	0.090*	
C48	0.8976 (13)	0.6037 (14)	0.3730 (8)	0.086 (4)	
H48A	0.9371	0.6693	0.3788	0.130*	
H48B	0.8981	0.5985	0.4161	0.130*	
H48C	0.8184	0.6056	0.3231	0.130*	
C54	0.6715 (11)	0.3223 (13)	0.0054 (6)	0.077 (4)	
H54A	0.6260	0.2582	−0.0236	0.116*	
H54B	0.6894	0.3593	−0.0232	0.116*	
H54C	0.6278	0.3701	0.0113	0.116*	
C55	0.7803 (10)	0.5459 (7)	0.8201 (7)	0.068 (3)	
H55	0.8067	0.5452	0.7926	0.082*	
C56	0.6333 (15)	0.5697 (15)	0.8199 (11)	0.143 (6)	
H56A	0.6947	0.5413	0.8724	0.214*	
H56B	0.5651	0.5248	0.7931	0.214*	
H56C	0.6152	0.6430	0.8238	0.214*	
C57	0.5914 (11)	0.5943 (11)	0.6963 (6)	0.086 (4)	
H57A	0.6356	0.6161	0.6818	0.129*	
H57B	0.5403	0.6527	0.6850	0.129*	
H57C	0.5458	0.5308	0.6662	0.129*	
C58	0.7778 (10)	−0.1278 (9)	0.3182 (8)	0.080 (3)	
H58	0.7912	−0.0906	0.2886	0.096*	
C59	0.6513 (12)	−0.2339 (13)	0.3288 (9)	0.112 (5)	
H59A	0.7138	−0.2215	0.3849	0.168*	
H59B	0.6453	−0.3106	0.3178	0.168*	
H59C	0.5793	−0.2083	0.3131	0.168*	
C60	0.5787 (17)	−0.1630 (16)	0.1896 (12)	0.161 (8)	
H60A	0.5966	−0.1041	0.1708	0.241*	
H60B	0.5051	−0.1492	0.1755	0.241*	
H60C	0.5741	−0.2301	0.1654	0.241*	

### Atomic displacement parameters (Å^2^)

**Table:**

	*U*^11^	*U*^22^	*U*^33^	*U*^12^	*U*^13^	*U*^23^
Br1	0.0544 (7)	0.1203 (13)	0.0898 (9)	0.0469 (8)	0.0437 (7)	0.0423 (9)
Br2	0.0536 (8)	0.1371 (17)	0.1670 (18)	−0.0138 (9)	0.0445 (11)	−0.0857 (15)
Zn1	0.0284 (4)	0.0361 (5)	0.0374 (5)	−0.0018 (5)	0.0184 (4)	−0.0056 (5)
Zn2	0.0540 (7)	0.0451 (7)	0.0582 (8)	0.0078 (6)	0.0185 (6)	0.0086 (7)
S1	0.0290 (10)	0.0372 (13)	0.0389 (12)	−0.0004 (10)	0.0163 (10)	−0.0066 (11)
S2	0.0691 (19)	0.0521 (18)	0.0518 (17)	0.0037 (16)	0.0185 (15)	0.0069 (15)
O1	0.045 (4)	0.105 (7)	0.063 (4)	0.007 (4)	0.033 (4)	−0.032 (5)
O2	0.029 (3)	0.059 (4)	0.028 (3)	0.010 (3)	0.011 (3)	−0.003 (3)
O3	0.073 (6)	0.074 (6)	0.112 (7)	−0.001 (5)	0.049 (6)	0.013 (6)
O4	0.072 (6)	0.077 (7)	0.112 (8)	0.009 (5)	0.048 (6)	0.027 (6)
O5	0.043 (3)	0.085 (5)	0.054 (4)	0.012 (3)	0.025 (3)	0.005 (4)
O6	0.086 (5)	0.081 (5)	0.052 (4)	−0.009 (4)	0.024 (4)	−0.009 (4)
N1	0.020 (3)	0.044 (4)	0.026 (3)	−0.004 (3)	0.009 (3)	−0.012 (3)
N2	0.031 (3)	0.034 (4)	0.033 (3)	0.002 (3)	0.017 (3)	−0.011 (3)
N3	0.032 (4)	0.052 (5)	0.044 (4)	0.011 (4)	0.023 (4)	−0.008 (4)
N4	0.041 (4)	0.030 (4)	0.030 (4)	0.000 (3)	0.020 (4)	−0.003 (3)
C16	0.033 (4)	0.045 (6)	0.047 (5)	0.002 (4)	0.018 (4)	0.005 (5)
C17	0.081 (8)	0.039 (6)	0.053 (6)	0.002 (6)	0.042 (7)	0.007 (5)
C18	0.087 (8)	0.036 (6)	0.079 (7)	−0.005 (6)	0.063 (7)	0.013 (6)
C19	0.045 (5)	0.051 (6)	0.059 (6)	−0.006 (5)	0.030 (5)	0.007 (5)
C20	0.034 (4)	0.026 (4)	0.050 (5)	−0.003 (4)	0.026 (4)	−0.004 (4)
N5	0.050 (4)	0.035 (4)	0.040 (4)	−0.003 (4)	0.032 (4)	−0.005 (4)
C22	0.038 (4)	0.042 (5)	0.040 (5)	0.001 (4)	0.026 (4)	−0.001 (4)
C23	0.039 (5)	0.042 (5)	0.048 (5)	−0.013 (4)	0.028 (5)	−0.006 (5)
C24	0.062 (6)	0.040 (5)	0.041 (5)	0.008 (5)	0.025 (5)	0.002 (5)
C25	0.063 (6)	0.095 (10)	0.027 (5)	−0.020 (6)	0.024 (5)	0.008 (6)
C26	0.067 (6)	0.033 (5)	0.048 (5)	−0.008 (5)	0.041 (5)	−0.007 (5)
N6	0.089 (7)	0.041 (6)	0.079 (7)	−0.003 (5)	0.043 (6)	−0.009 (5)
N7	0.066 (6)	0.066 (8)	0.077 (7)	0.006 (6)	0.028 (6)	−0.007 (6)
N8	0.064 (6)	0.071 (7)	0.055 (6)	0.032 (5)	0.027 (5)	0.013 (5)
N9	0.056 (5)	0.048 (6)	0.048 (5)	0.002 (4)	0.024 (5)	0.003 (5)
C43	0.129 (11)	0.053 (8)	0.046 (7)	0.009 (8)	0.047 (8)	0.008 (6)
C44	0.084 (9)	0.070 (9)	0.052 (7)	−0.023 (7)	0.041 (7)	−0.014 (7)
C45	0.053 (6)	0.054 (7)	0.059 (6)	−0.009 (5)	0.028 (5)	0.005 (6)
C46	0.062 (6)	0.041 (6)	0.042 (5)	0.005 (5)	0.031 (5)	0.009 (5)
C47	0.049 (5)	0.054 (6)	0.034 (5)	0.006 (5)	0.023 (5)	0.006 (5)
N10	0.044 (4)	0.040 (4)	0.050 (5)	0.004 (4)	0.019 (4)	0.000 (5)
C49	0.048 (5)	0.043 (6)	0.047 (6)	0.002 (5)	0.025 (5)	0.005 (5)
C50	0.066 (7)	0.072 (8)	0.035 (6)	−0.006 (6)	0.022 (6)	0.001 (6)
C51	0.065 (7)	0.091 (10)	0.045 (6)	0.018 (7)	0.034 (6)	0.013 (7)
C52	0.089 (8)	0.035 (6)	0.054 (6)	−0.014 (5)	0.041 (6)	−0.005 (5)
C53	0.062 (7)	0.067 (8)	0.063 (7)	−0.008 (6)	0.030 (6)	−0.021 (7)
N11	0.043 (4)	0.090 (6)	0.050 (5)	0.020 (4)	0.018 (4)	0.002 (4)
N12	0.086 (7)	0.133 (8)	0.073 (6)	−0.014 (6)	0.045 (5)	−0.015 (6)
C1	0.076 (8)	0.088 (9)	0.102 (9)	0.009 (7)	0.064 (8)	−0.035 (8)
C2	0.050 (5)	0.057 (7)	0.053 (6)	−0.004 (5)	0.038 (5)	−0.005 (5)
C3	0.044 (5)	0.080 (8)	0.067 (7)	0.022 (5)	0.039 (5)	0.012 (6)
C4	0.045 (5)	0.042 (5)	0.075 (7)	0.024 (4)	0.038 (6)	0.014 (5)
C5	0.040 (5)	0.058 (7)	0.049 (6)	0.020 (5)	0.022 (5)	0.020 (5)
C6	0.024 (4)	0.042 (5)	0.048 (5)	0.011 (4)	0.021 (4)	0.009 (4)
C7	0.054 (6)	0.033 (5)	0.077 (7)	−0.018 (4)	0.048 (6)	−0.025 (5)
C8	0.045 (5)	0.042 (6)	0.034 (4)	0.002 (4)	0.024 (4)	−0.003 (4)
C9	0.043 (5)	0.033 (5)	0.053 (5)	−0.014 (4)	0.036 (5)	−0.019 (4)
C10	0.038 (4)	0.041 (5)	0.048 (5)	0.001 (4)	0.028 (4)	−0.014 (4)
C11	0.055 (9)	0.027 (6)	0.060 (9)	−0.003 (6)	0.044 (8)	−0.006 (6)
C12	0.051 (10)	0.091 (14)	0.089 (14)	−0.009 (8)	0.039 (10)	−0.058 (11)
C13	0.087 (10)	0.061 (12)	0.072 (10)	0.005 (9)	0.044 (9)	−0.035 (10)
C14	0.052 (9)	0.061 (10)	0.058 (10)	−0.004 (7)	0.033 (9)	−0.021 (8)
C15	0.046 (8)	0.037 (8)	0.050 (8)	0.003 (6)	0.032 (8)	−0.004 (7)
C10'	0.038 (4)	0.041 (5)	0.048 (5)	0.001 (4)	0.028 (4)	−0.014 (4)
C11'	0.046 (8)	0.037 (8)	0.050 (8)	0.003 (6)	0.032 (8)	−0.004 (7)
C12'	0.052 (9)	0.061 (10)	0.058 (10)	−0.004 (7)	0.033 (9)	−0.021 (8)
C13'	0.087 (10)	0.061 (12)	0.072 (10)	0.005 (9)	0.044 (9)	−0.035 (10)
C14'	0.051 (10)	0.091 (14)	0.089 (14)	−0.009 (8)	0.039 (10)	−0.058 (11)
C15'	0.055 (9)	0.027 (6)	0.060 (9)	−0.003 (6)	0.044 (8)	−0.006 (6)
C21	0.091 (9)	0.054 (7)	0.096 (10)	−0.030 (7)	0.050 (8)	0.015 (7)
C27	0.104 (11)	0.110 (14)	0.048 (8)	−0.024 (11)	0.033 (8)	−0.005 (9)
C28	0.101 (14)	0.23 (3)	0.19 (2)	−0.026 (17)	0.101 (16)	0.03 (2)
C29	0.043 (6)	0.047 (7)	0.148 (13)	0.001 (6)	0.046 (8)	0.001 (9)
C30	0.059 (7)	0.045 (7)	0.132 (13)	0.021 (6)	0.041 (8)	0.003 (8)
C31	0.027 (5)	0.103 (12)	0.098 (10)	0.010 (7)	0.017 (6)	−0.043 (9)
C32	0.044 (7)	0.089 (11)	0.090 (10)	0.024 (7)	0.020 (7)	−0.013 (9)
C33	0.066 (7)	0.042 (7)	0.081 (9)	0.017 (6)	0.027 (7)	0.010 (6)
C34	0.028 (5)	0.053 (7)	0.066 (7)	−0.009 (5)	0.003 (5)	−0.017 (6)
C35	0.041 (6)	0.063 (8)	0.071 (8)	0.013 (6)	0.002 (6)	−0.001 (7)
C36	0.045 (5)	0.052 (7)	0.047 (6)	−0.002 (5)	0.017 (5)	−0.016 (5)
C37	0.059 (6)	0.083 (9)	0.038 (5)	0.029 (6)	0.027 (5)	−0.003 (6)
C38	0.082 (8)	0.082 (10)	0.071 (8)	0.018 (7)	0.047 (7)	0.008 (7)
C39	0.120 (12)	0.073 (10)	0.095 (10)	0.007 (8)	0.077 (10)	0.002 (8)
C40	0.095 (10)	0.119 (14)	0.054 (7)	0.048 (10)	0.043 (8)	0.017 (8)
C41	0.069 (7)	0.101 (11)	0.074 (8)	0.025 (7)	0.041 (7)	0.006 (7)
C42	0.052 (6)	0.091 (9)	0.061 (6)	0.034 (6)	0.029 (5)	0.016 (7)
C48	0.106 (10)	0.095 (11)	0.080 (9)	−0.015 (9)	0.070 (9)	−0.021 (8)
C54	0.067 (8)	0.074 (10)	0.040 (6)	0.004 (7)	0.014 (6)	0.005 (6)
C55	0.064 (6)	0.072 (6)	0.057 (5)	0.006 (5)	0.035 (5)	−0.008 (5)
C56	0.114 (9)	0.177 (11)	0.142 (10)	0.006 (7)	0.087 (8)	0.006 (8)
C57	0.072 (6)	0.103 (7)	0.050 (5)	0.017 (5)	0.026 (5)	0.001 (5)
C58	0.066 (6)	0.075 (7)	0.064 (6)	−0.020 (5)	0.028 (5)	−0.006 (5)
C59	0.099 (7)	0.119 (8)	0.105 (8)	−0.025 (6)	0.061 (6)	−0.007 (6)
C60	0.154 (11)	0.165 (12)	0.130 (10)	−0.038 (8)	0.079 (8)	0.015 (8)

### Geometric parameters (Å, º)

**Table:**

Br1—C4	1.877 (4)	C1—H1B	0.9600
Br2—C31	1.908 (5)	C1—H1C	0.9600
Zn1—O2	1.987 (6)	C2—C3	1.3900
Zn1—N4	2.088 (4)	C2—C7	1.3900
Zn1—N1	2.124 (6)	C3—C4	1.3900
Zn1—N5	2.134 (4)	C3—H3A	0.9300
Zn1—S1	2.353 (2)	C4—C5	1.3900
Zn2—O4	1.970 (9)	C5—C6	1.3900
Zn2—N6	2.063 (11)	C5—H5	0.9300
Zn2—N9	2.077 (5)	C6—C7	1.3900
Zn2—N10	2.124 (4)	C6—C8	1.459 (10)
Zn2—S2	2.346 (4)	C8—H8A	0.9300
S1—C9	1.765 (10)	C10—C11	1.3900
S2—C36	1.720 (11)	C10—C15	1.3900
O1—C2	1.351 (8)	C11—C12	1.3900
O1—C1	1.441 (12)	C11—H11	0.9300
O2—C7	1.364 (7)	C12—C13	1.3900
O3—C29	1.379 (12)	C12—H12	0.9300
O3—C28	1.40 (2)	C13—C14	1.3900
O4—C34	1.330 (11)	C13—H13	0.9300
O5—C55	1.239 (13)	C14—C15	1.3900
O6—C58	1.218 (14)	C14—H14	0.9300
N1—C8	1.246 (11)	C15—H15	0.9300
N1—N2	1.388 (9)	C10'—C11'	1.3900
N2—C9	1.251 (11)	C10'—C15'	1.3900
N3—C9	1.401 (11)	C11'—C12'	1.3900
N3—C10'	1.409 (10)	C11'—H11'	0.9300
N3—C10	1.407 (10)	C12'—C13'	1.3900
N3—H3	0.8800	C12'—H12'	0.9300
N3—H3'	0.8800	C13'—C14'	1.3900
N4—C16	1.3900	C13'—H13'	0.9300
N4—C20	1.3900	C14'—C15'	1.3900
C16—C17	1.3900	C14'—H14'	0.9300
C16—H16	0.9300	C15'—H15'	0.9300
C17—C18	1.3900	C21—H21A	0.9600
C17—H17	0.9300	C21—H21B	0.9600
C18—C19	1.3900	C21—H21C	0.9600
C18—C21	1.527 (11)	C27—H27A	0.9600
C19—C20	1.3900	C27—H27B	0.9600
C19—H19	0.9300	C27—H27C	0.9600
C20—C22	1.408 (5)	C28—H28A	0.9600
N5—C22	1.3900	C28—H28B	0.9600
N5—C26	1.3900	C28—H28C	0.9600
C22—C23	1.3900	C29—C30	1.3900
C23—C24	1.3900	C29—C34	1.3900
C23—H23	0.9300	C30—C31	1.3900
C24—C25	1.3900	C30—H30	0.9300
C24—C27	1.493 (14)	C31—C32	1.3900
C25—C26	1.3900	C32—C33	1.3900
C25—H25	0.9300	C32—H32	0.9300
C26—H26	0.9300	C33—C34	1.3900
N6—C35	1.368 (16)	C33—C35	1.443 (17)
N6—N7	1.403 (15)	C35—H35	0.9300
N7—C36	1.362 (15)	C37—C38	1.3900
N8—C36	1.347 (16)	C37—C42	1.3900
N8—C37	1.443 (11)	C38—C39	1.3900
N8—H8	0.8800	C38—H38	0.9300
N9—C43	1.3900	C39—C40	1.3900
N9—C47	1.3900	C39—H39	0.9300
C43—C44	1.3900	C40—C41	1.3900
C43—H43	0.9300	C40—H40	0.9300
C44—C45	1.3900	C41—C42	1.3900
C44—H44	0.9300	C41—H41	0.9300
C45—C46	1.3900	C42—H42	0.9300
C45—C48	1.444 (15)	C48—H48A	0.9600
C46—C47	1.3900	C48—H48B	0.9600
C46—H46	0.9300	C48—H48C	0.9600
C47—C49	1.418 (6)	C54—H54A	0.9600
N10—C49	1.3900	C54—H54B	0.9600
N10—C53	1.3900	C54—H54C	0.9600
C49—C50	1.3900	C55—H55	0.9300
C50—C51	1.3900	C56—H56A	0.9600
C50—H50	0.9300	C56—H56B	0.9600
C51—C52	1.3900	C56—H56C	0.9600
C51—C54	1.534 (12)	C57—H57A	0.9600
C52—C53	1.3900	C57—H57B	0.9600
C52—H52	0.9300	C57—H57C	0.9600
C53—H53	0.9300	C58—H58	0.9300
N11—C55	1.333 (14)	C59—H59A	0.9600
N11—C56	1.339 (17)	C59—H59B	0.9600
N11—C57	1.407 (14)	C59—H59C	0.9600
N12—C58	1.301 (16)	C60—H60A	0.9600
N12—C59	1.528 (18)	C60—H60B	0.9600
N12—C60	1.56 (2)	C60—H60C	0.9600
C1—H1A	0.9600		
			
O2—Zn1—N4	103.2 (3)	O2—C7—C2	116.8 (5)
O2—Zn1—N1	87.7 (2)	C6—C7—C2	120.0
N4—Zn1—N1	104.9 (3)	N1—C8—C6	128.3 (8)
O2—Zn1—N5	93.9 (2)	N1—C8—H8A	115.9
N4—Zn1—N5	78.0 (2)	C6—C8—H8A	115.9
N1—Zn1—N5	176.3 (3)	N2—C9—N3	119.1 (8)
O2—Zn1—S1	148.2 (2)	N2—C9—S1	129.3 (6)
N4—Zn1—S1	108.48 (15)	N3—C9—S1	111.4 (7)
N1—Zn1—S1	82.0 (2)	C11—C10—C15	120.0
N5—Zn1—S1	94.97 (17)	C11—C10—N3	115.3 (14)
O4—Zn2—N6	88.1 (4)	C15—C10—N3	124.6 (14)
O4—Zn2—N9	98.8 (4)	C12—C11—C10	120.0
N6—Zn2—N9	106.6 (3)	C12—C11—H11	120.0
O4—Zn2—N10	93.8 (3)	C10—C11—H11	120.0
N6—Zn2—N10	174.8 (4)	C13—C12—C11	120.0
N9—Zn2—N10	77.9 (2)	C13—C12—H12	120.0
O4—Zn2—S2	151.3 (4)	C11—C12—H12	120.0
N6—Zn2—S2	81.5 (3)	C12—C13—C14	120.0
N9—Zn2—S2	109.7 (2)	C12—C13—H13	120.0
N10—Zn2—S2	94.6 (2)	C14—C13—H13	120.0
C9—S1—Zn1	93.8 (3)	C15—C14—C13	120.0
C36—S2—Zn2	96.7 (4)	C15—C14—H14	120.0
C2—O1—C1	120.0 (9)	C13—C14—H14	120.0
C7—O2—Zn1	126.8 (4)	C14—C15—C10	120.0
C29—O3—C28	119.4 (13)	C14—C15—H15	120.0
C34—O4—Zn2	128.5 (8)	C10—C15—H15	120.0
C8—N1—N2	117.1 (7)	C11'—C10'—C15'	120.0
C8—N1—Zn1	123.4 (6)	C11'—C10'—N3	117.3 (15)
N2—N1—Zn1	119.5 (5)	C15'—C10'—N3	122.7 (15)
C9—N2—N1	114.2 (6)	C12'—C11'—C10'	120.0
C9—N3—C10'	129.2 (11)	C12'—C11'—H11'	120.0
C9—N3—C10	130.1 (11)	C10'—C11'—H11'	120.0
C10'—N3—C10	3.4 (16)	C11'—C12'—C13'	120.0
C9—N3—H3	115.0	C11'—C12'—H12'	120.0
C10'—N3—H3	115.8	C13'—C12'—H12'	120.0
C10—N3—H3	115.0	C14'—C13'—C12'	120.0
C9—N3—H3'	115.4	C14'—C13'—H13'	120.0
C10'—N3—H3'	115.4	C12'—C13'—H13'	120.0
C10—N3—H3'	114.4	C15'—C14'—C13'	120.0
C16—N4—C20	120.0	C15'—C14'—H14'	120.0
C16—N4—Zn1	125.1 (3)	C13'—C14'—H14'	120.0
C20—N4—Zn1	114.9 (3)	C14'—C15'—C10'	120.0
C17—C16—N4	120.0	C14'—C15'—H15'	120.0
C17—C16—H16	120.0	C10'—C15'—H15'	120.0
N4—C16—H16	120.0	C18—C21—H21A	109.5
C16—C17—C18	120.0	C18—C21—H21B	109.5
C16—C17—H17	120.0	H21A—C21—H21B	109.5
C18—C17—H17	120.0	C18—C21—H21C	109.5
C17—C18—C19	120.0	H21A—C21—H21C	109.5
C17—C18—C21	119.4 (7)	H21B—C21—H21C	109.5
C19—C18—C21	120.4 (7)	C24—C27—H27A	109.5
C20—C19—C18	120.0	C24—C27—H27B	109.5
C20—C19—H19	120.0	H27A—C27—H27B	109.5
C18—C19—H19	120.0	C24—C27—H27C	109.5
C19—C20—N4	120.0	H27A—C27—H27C	109.5
C19—C20—C22	123.1 (4)	H27B—C27—H27C	109.5
N4—C20—C22	116.9 (4)	O3—C28—H28A	109.5
C22—N5—C26	120.0	O3—C28—H28B	109.5
C22—N5—Zn1	113.5 (3)	H28A—C28—H28B	109.5
C26—N5—Zn1	126.1 (3)	O3—C28—H28C	109.5
C23—C22—N5	120.0	H28A—C28—H28C	109.5
C23—C22—C20	123.5 (4)	H28B—C28—H28C	109.5
N5—C22—C20	116.5 (4)	O3—C29—C30	121.3 (7)
C22—C23—C24	120.0	O3—C29—C34	118.7 (7)
C22—C23—H23	120.0	C30—C29—C34	120.0
C24—C23—H23	120.0	C29—C30—C31	120.0
C25—C24—C23	120.0	C29—C30—H30	120.0
C25—C24—C27	118.4 (8)	C31—C30—H30	120.0
C23—C24—C27	121.6 (8)	C32—C31—C30	120.0
C24—C25—C26	120.0	C32—C31—Br2	118.7 (6)
C24—C25—H25	120.0	C30—C31—Br2	121.3 (6)
C26—C25—H25	120.0	C31—C32—C33	120.0
C25—C26—N5	120.0	C31—C32—H32	120.0
C25—C26—H26	120.0	C33—C32—H32	120.0
N5—C26—H26	120.0	C34—C33—C32	120.0
C35—N6—N7	110.1 (11)	C34—C33—C35	126.8 (8)
C35—N6—Zn2	126.4 (10)	C32—C33—C35	113.2 (8)
N7—N6—Zn2	122.7 (8)	O4—C34—C33	123.9 (7)
C36—N7—N6	110.8 (10)	O4—C34—C29	115.9 (7)
C36—N8—C37	131.6 (10)	C33—C34—C29	120.0
C36—N8—H8	114.2	N6—C35—C33	120.5 (12)
C37—N8—H8	114.2	N6—C35—H35	119.7
C43—N9—C47	120.0	C33—C35—H35	119.7
C43—N9—Zn2	124.5 (3)	N8—C36—N7	117.3 (11)
C47—N9—Zn2	115.3 (3)	N8—C36—S2	116.2 (8)
C44—C43—N9	120.0	N7—C36—S2	126.3 (10)
C44—C43—H43	120.0	C38—C37—C42	120.0
N9—C43—H43	120.0	C38—C37—N8	116.8 (7)
C45—C44—C43	120.0	C42—C37—N8	123.0 (7)
C45—C44—H44	120.0	C39—C38—C37	120.0
C43—C44—H44	120.0	C39—C38—H38	120.0
C44—C45—C46	120.0	C37—C38—H38	120.0
C44—C45—C48	121.7 (7)	C38—C39—C40	120.0
C46—C45—C48	118.3 (7)	C38—C39—H39	120.0
C47—C46—C45	120.0	C40—C39—H39	120.0
C47—C46—H46	120.0	C41—C40—C39	120.0
C45—C46—H46	120.0	C41—C40—H40	120.0
C46—C47—N9	120.0	C39—C40—H40	120.0
C46—C47—C49	123.5 (5)	C40—C41—C42	120.0
N9—C47—C49	116.5 (5)	C40—C41—H41	120.0
C49—N10—C53	120.0	C42—C41—H41	120.0
C49—N10—Zn2	114.0 (3)	C41—C42—C37	120.0
C53—N10—Zn2	125.7 (3)	C41—C42—H42	120.0
N10—C49—C50	120.0	C37—C42—H42	120.0
N10—C49—C47	115.7 (5)	C45—C48—H48A	109.5
C50—C49—C47	124.2 (5)	C45—C48—H48B	109.5
C49—C50—C51	120.0	H48A—C48—H48B	109.5
C49—C50—H50	120.0	C45—C48—H48C	109.5
C51—C50—H50	120.0	H48A—C48—H48C	109.5
C50—C51—C52	120.0	H48B—C48—H48C	109.5
C50—C51—C54	118.4 (7)	C51—C54—H54A	109.5
C52—C51—C54	121.5 (7)	C51—C54—H54B	109.5
C53—C52—C51	120.0	H54A—C54—H54B	109.5
C53—C52—H52	120.0	C51—C54—H54C	109.5
C51—C52—H52	120.0	H54A—C54—H54C	109.5
C52—C53—N10	120.0	H54B—C54—H54C	109.5
C52—C53—H53	120.0	O5—C55—N11	125.5 (10)
N10—C53—H53	120.0	O5—C55—H55	117.3
C55—N11—C56	115.6 (11)	N11—C55—H55	117.3
C55—N11—C57	122.3 (10)	N11—C56—H56A	109.5
C56—N11—C57	122.1 (11)	N11—C56—H56B	109.5
C58—N12—C59	121.0 (12)	H56A—C56—H56B	109.5
C58—N12—C60	119.7 (13)	N11—C56—H56C	109.5
C59—N12—C60	119.3 (12)	H56A—C56—H56C	109.5
O1—C1—H1A	109.5	H56B—C56—H56C	109.5
O1—C1—H1B	109.5	N11—C57—H57A	109.5
H1A—C1—H1B	109.5	N11—C57—H57B	109.5
O1—C1—H1C	109.5	H57A—C57—H57B	109.5
H1A—C1—H1C	109.5	N11—C57—H57C	109.5
H1B—C1—H1C	109.5	H57A—C57—H57C	109.5
O1—C2—C3	124.2 (5)	H57B—C57—H57C	109.5
O1—C2—C7	115.8 (5)	O6—C58—N12	120.6 (13)
C3—C2—C7	120.0	O6—C58—H58	119.7
C2—C3—C4	120.0	N12—C58—H58	119.7
C2—C3—H3A	120.0	N12—C59—H59A	109.5
C4—C3—H3A	120.0	N12—C59—H59B	109.5
C5—C4—C3	120.0	H59A—C59—H59B	109.5
C5—C4—Br1	121.3 (3)	N12—C59—H59C	109.5
C3—C4—Br1	118.7 (3)	H59A—C59—H59C	109.5
C4—C5—C6	120.0	H59B—C59—H59C	109.5
C4—C5—H5	120.0	N12—C60—H60A	109.5
C6—C5—H5	120.0	N12—C60—H60B	109.5
C5—C6—C7	120.0	H60A—C60—H60B	109.5
C5—C6—C8	116.6 (5)	N12—C60—H60C	109.5
C7—C6—C8	123.3 (5)	H60A—C60—H60C	109.5
O2—C7—C6	123.2 (4)	H60B—C60—H60C	109.5
			
O2—Zn1—S1—C9	−80.2 (4)	C46—C47—C49—N10	179.3 (4)
N4—Zn1—S1—C9	95.2 (3)	N9—C47—C49—N10	−3.0 (7)
N1—Zn1—S1—C9	−7.9 (3)	C46—C47—C49—C50	1.7 (8)
N5—Zn1—S1—C9	174.3 (3)	N9—C47—C49—C50	179.4 (4)
O4—Zn2—S2—C36	77.1 (8)	N10—C49—C50—C51	0.0
N6—Zn2—S2—C36	7.2 (5)	C47—C49—C50—C51	177.6 (7)
N9—Zn2—S2—C36	−97.5 (4)	C49—C50—C51—C52	0.0
N10—Zn2—S2—C36	−176.2 (4)	C49—C50—C51—C54	176.8 (9)
N4—Zn1—O2—C7	−75.8 (7)	C50—C51—C52—C53	0.0
N1—Zn1—O2—C7	29.0 (7)	C54—C51—C52—C53	−176.7 (9)
N5—Zn1—O2—C7	−154.4 (7)	C51—C52—C53—N10	0.0
S1—Zn1—O2—C7	99.7 (7)	C49—N10—C53—C52	0.0
N6—Zn2—O4—C34	−23.1 (10)	Zn2—N10—C53—C52	174.1 (5)
N9—Zn2—O4—C34	83.4 (10)	C1—O1—C2—C3	13.5 (13)
N10—Zn2—O4—C34	161.8 (10)	C1—O1—C2—C7	−169.2 (8)
S2—Zn2—O4—C34	−91.4 (12)	O1—C2—C3—C4	177.1 (8)
O2—Zn1—N1—C8	−21.4 (8)	C7—C2—C3—C4	0.0
N4—Zn1—N1—C8	81.7 (8)	C2—C3—C4—C5	0.0
S1—Zn1—N1—C8	−171.2 (8)	C2—C3—C4—Br1	178.8 (5)
O2—Zn1—N1—N2	159.4 (6)	C3—C4—C5—C6	0.0
N4—Zn1—N1—N2	−97.6 (6)	Br1—C4—C5—C6	−178.8 (5)
S1—Zn1—N1—N2	9.5 (6)	C4—C5—C6—C7	0.0
C8—N1—N2—C9	174.4 (9)	C4—C5—C6—C8	−176.4 (7)
Zn1—N1—N2—C9	−6.2 (9)	Zn1—O2—C7—C6	−22.1 (9)
O2—Zn1—N4—C16	88.1 (4)	Zn1—O2—C7—C2	158.2 (5)
N1—Zn1—N4—C16	−3.1 (4)	C5—C6—C7—O2	−179.7 (7)
N5—Zn1—N4—C16	179.3 (4)	C8—C6—C7—O2	−3.5 (9)
S1—Zn1—N4—C16	−89.4 (3)	C5—C6—C7—C2	0.0
O2—Zn1—N4—C20	−92.8 (3)	C8—C6—C7—C2	176.2 (7)
N1—Zn1—N4—C20	176.0 (3)	O1—C2—C7—O2	2.3 (8)
N5—Zn1—N4—C20	−1.6 (3)	C3—C2—C7—O2	179.7 (7)
S1—Zn1—N4—C20	89.7 (3)	O1—C2—C7—C6	−177.4 (8)
C20—N4—C16—C17	0.0	C3—C2—C7—C6	0.0
Zn1—N4—C16—C17	179.1 (4)	N2—N1—C8—C6	−173.8 (8)
N4—C16—C17—C18	0.0	Zn1—N1—C8—C6	6.9 (14)
C16—C17—C18—C19	0.0	C5—C6—C8—N1	−172.7 (9)
C16—C17—C18—C21	−174.2 (9)	C7—C6—C8—N1	11.0 (13)
C17—C18—C19—C20	0.0	N1—N2—C9—N3	−178.5 (7)
C21—C18—C19—C20	174.1 (9)	N1—N2—C9—S1	−3.7 (12)
C18—C19—C20—N4	0.0	C10'—N3—C9—N2	15.3 (17)
C18—C19—C20—C22	−178.4 (6)	C10—N3—C9—N2	19.6 (17)
C16—N4—C20—C19	0.0	C10'—N3—C9—S1	−160.3 (12)
Zn1—N4—C20—C19	−179.2 (4)	C10—N3—C9—S1	−156.0 (12)
C16—N4—C20—C22	178.5 (6)	Zn1—S1—C9—N2	9.6 (9)
Zn1—N4—C20—C22	−0.7 (5)	Zn1—S1—C9—N3	−175.2 (6)
O2—Zn1—N5—C22	106.4 (3)	C9—N3—C10—C11	−175.4 (13)
N4—Zn1—N5—C22	3.7 (3)	C15—C10—C11—C12	0.0
S1—Zn1—N5—C22	−104.2 (3)	N3—C10—C11—C12	−176.7 (17)
O2—Zn1—N5—C26	−81.3 (4)	C10—C11—C12—C13	0.0
N4—Zn1—N5—C26	176.0 (4)	C11—C12—C13—C14	0.0
S1—Zn1—N5—C26	68.2 (3)	C12—C13—C14—C15	0.0
C26—N5—C22—C23	0.0	C13—C14—C15—C10	0.0
Zn1—N5—C22—C23	172.8 (4)	C11—C10—C15—C14	0.0
C26—N5—C22—C20	−178.2 (5)	N3—C10—C15—C14	176.3 (19)
Zn1—N5—C22—C20	−5.3 (5)	C9—N3—C10'—C11'	171.4 (12)
C19—C20—C22—C23	4.4 (6)	C15'—C10'—C11'—C12'	0.0
N4—C20—C22—C23	−174.0 (4)	N3—C10'—C11'—C12'	−179.5 (17)
C19—C20—C22—N5	−177.5 (3)	C10'—C11'—C12'—C13'	0.0
N4—C20—C22—N5	4.1 (6)	C11'—C12'—C13'—C14'	0.0
N5—C22—C23—C24	0.0	C12'—C13'—C14'—C15'	0.0
C20—C22—C23—C24	178.0 (6)	C13'—C14'—C15'—C10'	0.0
C22—C23—C24—C25	0.0	C11'—C10'—C15'—C14'	0.0
C22—C23—C24—C27	179.9 (10)	N3—C10'—C15'—C14'	179.4 (18)
C23—C24—C25—C26	0.0	C28—O3—C29—C30	−12.1 (17)
C27—C24—C25—C26	−179.9 (10)	C28—O3—C29—C34	168.7 (14)
C24—C25—C26—N5	0.0	O3—C29—C30—C31	−179.2 (9)
C22—N5—C26—C25	0.0	C34—C29—C30—C31	0.0
Zn1—N5—C26—C25	−171.9 (4)	C29—C30—C31—C32	0.0
O4—Zn2—N6—C35	23.3 (12)	C29—C30—C31—Br2	−179.6 (6)
N9—Zn2—N6—C35	−75.4 (11)	C30—C31—C32—C33	0.0
S2—Zn2—N6—C35	176.5 (11)	Br2—C31—C32—C33	179.6 (6)
O4—Zn2—N6—N7	−167.5 (10)	C31—C32—C33—C34	0.0
N9—Zn2—N6—N7	93.9 (9)	C31—C32—C33—C35	177.5 (9)
S2—Zn2—N6—N7	−14.3 (9)	Zn2—O4—C34—C33	12.8 (14)
C35—N6—N7—C36	−173.2 (11)	Zn2—O4—C34—C29	−162.2 (7)
Zn2—N6—N7—C36	16.0 (13)	C32—C33—C34—O4	−174.8 (10)
O4—Zn2—N9—C43	−88.2 (5)	C35—C33—C34—O4	8.0 (12)
N6—Zn2—N9—C43	2.5 (5)	C32—C33—C34—C29	0.0
N10—Zn2—N9—C43	179.8 (4)	C35—C33—C34—C29	−177.1 (10)
S2—Zn2—N9—C43	89.2 (4)	O3—C29—C34—O4	−5.5 (10)
O4—Zn2—N9—C47	97.4 (5)	C30—C29—C34—O4	175.2 (9)
N6—Zn2—N9—C47	−171.9 (4)	O3—C29—C34—C33	179.2 (9)
N10—Zn2—N9—C47	5.4 (4)	C30—C29—C34—C33	0.0
S2—Zn2—N9—C47	−85.2 (4)	N7—N6—C35—C33	176.1 (10)
C47—N9—C43—C44	0.0	Zn2—N6—C35—C33	−13.5 (18)
Zn2—N9—C43—C44	−174.1 (5)	C34—C33—C35—N6	−6.9 (16)
N9—C43—C44—C45	0.0	C32—C33—C35—N6	175.8 (10)
C43—C44—C45—C46	0.0	C37—N8—C36—N7	−24.5 (17)
C43—C44—C45—C48	179.1 (9)	C37—N8—C36—S2	160.5 (9)
C44—C45—C46—C47	0.0	N6—N7—C36—N8	178.0 (9)
C48—C45—C46—C47	−179.1 (8)	N6—N7—C36—S2	−7.6 (15)
C45—C46—C47—N9	0.0	Zn2—S2—C36—N8	172.4 (8)
C45—C46—C47—C49	177.6 (7)	Zn2—S2—C36—N7	−2.1 (11)
C43—N9—C47—C46	0.0	C36—N8—C37—C38	−173.7 (10)
Zn2—N9—C47—C46	174.6 (4)	C36—N8—C37—C42	1.4 (15)
C43—N9—C47—C49	−177.8 (6)	C42—C37—C38—C39	0.0
Zn2—N9—C47—C49	−3.2 (6)	N8—C37—C38—C39	175.2 (7)
O4—Zn2—N10—C49	−105.2 (5)	C37—C38—C39—C40	0.0
N9—Zn2—N10—C49	−6.9 (4)	C38—C39—C40—C41	0.0
S2—Zn2—N10—C49	102.3 (3)	C39—C40—C41—C42	0.0
O4—Zn2—N10—C53	80.4 (5)	C40—C41—C42—C37	0.0
N9—Zn2—N10—C53	178.7 (4)	C38—C37—C42—C41	0.0
S2—Zn2—N10—C53	−72.2 (4)	N8—C37—C42—C41	−174.9 (8)
C53—N10—C49—C50	0.0	C56—N11—C55—O5	−0.1 (4)
Zn2—N10—C49—C50	−174.8 (4)	C57—N11—C55—O5	179.4 (4)
C53—N10—C49—C47	−177.8 (6)	C59—N12—C58—O6	0.0 (3)
Zn2—N10—C49—C47	7.5 (6)	C60—N12—C58—O6	−179.8 (3)

### Hydrogen-bond geometry (Å, º)

**Table:**

*D*—H···*A*	*D*—H	H···*A*	*D*···*A*	*D*—H···*A*
N3—H3···O5	0.88	2.07	2.95 (1)	175
N8—H8···O6	0.88	2.07	2.95 (1)	172
